# The Law of Similars: Second Open Letter to an Inquiring Physician

**Published:** 1894-07

**Authors:** Edward Cranch


### THE LAW OF SIMILARS.

### SECOND OPEN LETTER TO AN INQUIRING PHYSICIAN.

You must, of course, have heard some of the preposterous statements afloat that are commonly offered as illustrations of the homœopathic doctrine of Similars. Some of them are almost too idle for thought to fasten upon, but as they are repeated to the thoughtless, they sometimes sound plausible; such as the following: "If you have taken too much Opium, you must take more to cure you, that's Homœopathy," or "you have eaten too big a dinner, eat a little more," or else, "you have had a fall, now take a beating," or again, "you are drunk, take another glass of whiskey," and so forth, with a laugh or a sneer at the "little pills," or the "water medicine," or the "faith cure," or the "moonshine," and "bottle-washing;" and so an end of argument, and the substitution of ridicule.

Doubtless ridicule is a potent weapon, and has deterred many a careless man who loves his ease from honest investigation, but you are not cut on that pattern; you inquire, and you are willing to try, because you are true to science.

In your studies of Hahnemann, you have perhaps come to that passage in his *Materia Medica Pura*, the preface to his third volume, a "Nota Bene" for his reviewers. In it he says to his opponents who wish to destroy his system: "This doctrine rests exclusively upon experience. Imitate its indications and you will find that they are true. * * * Take a case, note down all its perceptible symptoms in the manner taught in *The Organon* * * * apply that drug (if one has been yet proven) which shall be perfectly homœopathic to all the symptoms, the dose being as prescribed in *The Organon*, avoid all disturbing agencies, and if under these circumstances, the drug does not afford speedy and efficient help, then publish the failure to the world in a manner which shall make it impossible to gainsay the homœopathicity of the drug, and the correctness of your proceedings, and the author of Homœopathy will stand confounded and convicted."

This challege has gained for us, in its trial, many converts, some of them most distinguished for their zeal in the very cause they attempted to overthrow, and earnest provers of new drugs, as the surest means to be able to relieve disease and suffering.

No one has accepted the challenge and been able to injure Homœopathy by its means. Notice the language of Hahnemann, when he says, "This doctrine rests solely on experience" —yet it opposes much of what has elsewhere been called the lesson of experience. Here it is, as we pointed out in the first letter: all discoveries must be proven and established by experience, although partial or fallacious experience may seem to have pointed in the past another way. If the law is true, then all past experience and apparently conflicting experience can be analyzed in the light of the law, just as the facts that Ptolemy relied on to sustain his terrecentral planetary system, became, when newly analyzed, proofs and elucidations of Copernicus' discovery, of the heliocentral arrangement of worlds and moons.

Thus, in the introduction of *The Organon*, Hahnemann has collected hosts of instances where cases were reported, without law, and then by other references, he shows that all those curing drugs were capable of causing the very conditions that in other hands they had cured. For the law is exclusive, however its enemies and its weak defenders may err in their illustrations of supposed conflict with, and cure by, other supposed laws.

*No real drug cure ever took place or can take place, except by a drug capable of causing a condition similar to the one cured.*

The doubts, if any, are caused by limited observation; either the alleged cures by other methods were incorrectly reported, or they were only palliative suppressions, not cures at all. If a drug cures, or relieves, it will surely be found, on full proving, that like conditions can be caused by that very drug.

To instance some of the most common cases in old physic; Catnip, Anise, Peppermint, etc., relieve colic; if persisted in too long they create disturbances ending in colic; Opium causes sleep; its prolonged use brings on insomnia; Sulphur cures certain eruptions; its injudicious use will bring on similar ones;

Borax cleanses the mouth and cures thrush; its excessive use will produce fœtid ulcerations of the mucous membranes resembling thrush, or aphthæ; Salicylic acid cures rheumatism; its prolonged use causes a return, or its use in food as a preservative provokes fresh rheumatism; Nitrate of Silver cures sore eyes, who will not acknowledge that it can also cause them? Nitric acid heals sluggish ulcers; it will also cause similar ones; blisters deplete scrofulous glands; the glands around a deep blister will enlarge. Cold bathing invigorates, its injudicious use causes even fatal collapse; Strychnia helps many cases of paresis; dogs poisoned by it show paralysis before death; Calomel checks infantile diarrhœa, it is most frequently used as a purgative; Ergot checks hemorrhage, its prolonged use brings on the same; Soda in the stomach corrects acidity, its over-use provokes increased acidity; Turpentine arrests hemorrhage; it has often caused it. In the same way, every cure reported may be examined, and shown to have operated on similar lines. Take one of the most apparently dissimilar cases, the use of Iodine in hypertrophic enlargements, when every one knows that Iodine causes emaciation and wasting of tissue, even in the healthy; the reduction of tissue is either followed by dangerous symptoms showing that no real cure has taken place, or, if successful, the system has roused itself to healthy reaction such as follows sea-bathing and sea air, viz.: an increased appetite (one of the first effects of Iodine) and subsequent fattening, when the iodism is not strong enough to overcome the system. In such cases the *reaction* fixes the cure, and permits the system to escape unharmed from the first vicious assault of the drug.

Nature is ever kind, and general reaction from depressing influences, however at random they may be selected, will often effect a cure which is too often attributed to the wrong cause. The homœopathic doctrine asserts without fear of successful contradiction that vital reaction is the cause of every cure, and that no cure will ever be reached, if vital reaction is not allowed to assert itself.

It was vital reaction that cured in spite of bleedings, purgings, emetics, cauteries, blisters, lotions, and potions, and it was only because these things could cause sickness, that they ever succeeded in rousing the needed reaction. If the sick-making action was too strong, the patient succumbed, and then, no successful reaction taking place, the poor man either died and was buried, or else lingered in a wretched state of chronic disease and disorder.

Somehow, we are all too prone to say and hence to think, unguardedly, that the medicine, or the means employed to cure, really cured, forgetting that all such means can do is to rouse vital reaction, and guide it to a healthy issue.

From this fallacy comes the tacit rule of the older physic, "give all the patient can stand," as exemplified in the dose list; from a recognition of the vital force of the soul, ever trying to come to health in the body, comes the rule, or corollary of the homœopaths, "give the smallest dose that will cure."

Do not forget the main part of the law we claim, for it is true everywhere, viz.: *every action excites an opposite and equal reaction*, and this being true in metaphysics we shall expect this letter to excite an opposite reaction in the shape of doubts; but these may and should only create an appetite for investigation, which will in time remove doubts and establish the real truth. In our next we will say something about the practice of the law, or how to reap the surest benefits from its knowledge.

EDWARD CRANCH, M. D.

